# Integrated proteomic and metabolomic analysis reveals that rhodomyrtone reduces the capsule in *Streptococcus pneumoniae*

**DOI:** 10.1038/s41598-017-02996-3

**Published:** 2017-06-02

**Authors:** Watcharapong Mitsuwan, Alfonso Olaya-Abril, Mónica Calderón-Santiago, Irene Jiménez-Munguía, José Antonio González-Reyes, Feliciano Priego-Capote, Supayang P. Voravuthikunchai, Manuel J. Rodríguez-Ortega

**Affiliations:** 1 Department of Microbiology and Excellent Research Laboratory on Natural Products, Faculty of Science and Natural Product Research Center of Excellence Prince of Songkla University, Songkhla, Thailand; 2 Departamento de Bioquímica y Biología Molecular, Universidad de Córdoba; Campus de Excelencia Internacional CeiA3, Córdoba, Spain; 3 Departamento de Química Analítica, Universidad de Córdoba; Campus de Excelencia Internacional CeiA3, Córdoba, Spain; 4 Departamento de Biología Celular, Fisiología e Inmunología, Universidad de Córdoba; Campus de Excelencia Internacional CeiA3, Córdoba, Spain

## Abstract

The emergence of antibiotic-resistant pathogenic bacteria is a healthcare problem worldwide. We evaluated the antimicrobial activity of rhodomyrtone, an acylphloroglucinol present in *Rhodomyrtus tomentosa* leaves, against the human Gram-positive pathogen *Streptococcus pneumoniae*. The compound exhibited pronounced anti-pneumococcal activity against a broad collection of clinical isolates. We studied the effects at the molecular level by integrated proteomic and metabolomic analysis. The results revealed alterations in enzymes and metabolites involved in several metabolic pathways including amino acid biosynthesis, nucleic acid biosynthesis, glucid, and lipid metabolism. Notably, the levels of two enzymes (glycosyltransferase and UTP-glucose-1-phosphate uridylyltransferase) and three metabolites (UDP-glucose, UDP-glucuronic acid and UDP-N-acetyl-D-galactosamine) participating in the synthesis of the pneumococcal capsule clearly diminished in the bacterial cells exposed to rhodomyrtone. Rhodomyrtone-treated pneumococci significantly possessed less amount of capsule, as measured by a colorimetric assay and visualized by electron microscopy. These findings reveal the utility of combining proteomic and metabolomic analyses to provide insight into phenotypic features of *S. pneumoniae* treated with this potential novel antibiotic. This can lead to an alternative antibiotic for the treatment of *S. pneumoniae* infections, because of the growing concern regarding antimicrobial resistance.

## Introduction

*Streptococcus pneumoniae* (the pneumococcus) is a Gram-positive bacterium that colonizes in the human upper respiratory tract. Under appropriate conditions, this microorganism may become a leading cause of serious diseases. *Streptococcus pneumoniae* infections are responsible for high morbidity and mortality rates in humans worldwide. Actually, it is estimated that around one million children <5 years die every year because of pneumococcal disease, mainly in developing countries^1, 2^. The pathogen causes a variety of diseases ranging from local infections to serious life-threatening diseases including community-acquired pneumonia, meningitis, bronchitis, sinusitis, otitis media, and septicemia^3, 4^. The pneumococcus produces a protective surface structure known as capsule that is considered as the main virulence factor^5–7^. The capsule plays crucial roles in the pathogenesis including the bacterial capacity of avoiding phagocytosis and preventing complement activity^8^. Moreover, the capsule is also a major immunogen that serves as the basis for pneumococcal serotyping and the development of protective vaccines^9^.

During the last decades, the emergence of antimicrobial resistance in bacterial infections has become a major public health concern worldwide^10^. In particular, the pneumococcus is increasingly resistant to the most common clinically used drugs such as β-lactam antibiotics and macrolides^11, 12^. Therefore, there is a growing interest in alternative strategies to control pneumococcal infections. Medicinal plants have been used to treat bacterial infections due to the activities of their secondary metabolites. *Rhodomyrtus tomentosa* (Aiton) Hassk. is a flowering medicinal plant that belongs to the family Myrtaceae. The plant has significant value in traditional medicine for the treatment of dysentery, diarrhea, and urinary tract infections^13^. Previous studies of our research group have shown that *Rhodomyrtus tomentosa* ethanol extract possesses strong antibacterial activity against a wide range of Gram-positive bacteria^14, 15^. Interestingly, rhodomyrtone, an acylphloroglucinol derivative isolated from this plant species, has demonstrated remarkable antibacterial activity against important human pathogens including the pneumococcus^14^.

The effects of rhodomyrtone at molecular level have been studied in a few Gram-positive species. Proteomic analysis has revealed that rhodomyrtone affected the expression of several major classes of cellular proteins in methicillin-resistant *Staphylococcus aureus* (MRSA)^16^. In addition, transcriptome analysis has revealed that rhodomyrtone caused a significant modulation of gene expression, with induction of 64 genes and repression of 35 genes in MRSA^17^. Also, proteomic analysis of rhodomyrtone-treated *Streptococcus pyogenes* has shown that the compound affects the expression of streptococcal secreted and whole cell proteins. Most of the altered proteins were identified as enzymes associated with important pathways of the primary metabolism^18^. However, the antibacterial mechanism of the compound is still unknown.

The aim of this work was to study the antibacterial effect of rhodomyrtone on *S. pneumoniae*, and the changes induced at molecular level using proteomics and metabolomics. We have studied the response of two reference pneumococcal strains including the virulent, encapsulated strain TIGR4 and the non-encapsulated avirulent strain R6 in the presence of the purified compound, using proteomics and metabolomics. This may help to provide insight into the mechanism of action of the substance, to be used as a possible antibiotic for the treatment of *S. pneumoniae* infections. The proteomic and metabolomic analyses have revealed alterations in enzymes and metabolites involved in pneumococcal capsule synthesis, further confirmed by capsule quantification on several clinical isolates and visualized by electron microscopy. Our work reveals the utility of multi-omic approaches to contribute to the comprehension of the effects of drugs to treat infectious diseases.

## Results

### *In vitro* anti-pneumococcal activity of *Rhodomyrtus tomentosa* ethanol extract and rhodomyrtone

We tested the antibacterial activity of *Rhodomyrtus tomentosa* ethanol extract, purified rhodomyrtone, and synthetic rhodomyrtone against a collection of pediatric *S. pneumoniae* clinical isolates (Table S1) by assaying the minimal inhibitory and bactericidal concentrations. Table 1 shows the MIC_50/90_ and MBC_50/90_ values for the three testing molecules/extract against the 23 selected isolates, compared to one of the three reference strains used, and using erythromycin as a positive control. The MIC/MBC values of the ethanol extract ranged from 16 to 512 µg/ml. Both purified and synthetic rhodomyrtone demonstrated a markedly pronounced antibacterial activity with similar MIC and MBC values ranging from 0.125 to 4 µg/ml. The MIC and MBC values of the extract, purified rhodomyrtone, and synthetic rhodomyrtone against the reference strains were in the same range as those of the tested clinical isolates (Table S1).

**Table 1:** Minimal inhibitory concentration (MIC)_50/90_ and minimal bactericidal concentration (MBC)_50/90_ values of *Rhodomyrtus tomentosa* ethanol extract, purified rhodomyrtone, and synthetic rhodomyrtone against *Streptococcus pneumoniae* clinical isolates.

Antibacterial agents	Antibacterial activity (µg/ml)				
	*S. pneumoniae* clinical isolates				*S. pneumoniae* ATCC 700673
	MIC_50_/MBC_50_	MIC_90_/MBC_90_	MIC range	MBC range	MIC/MBC
Ethanol extract	64/128	128/512	16–256	16–512	32/128
Purified rhodomyrtone	0.50/1	2/4	0.125–4	0.125–4	0.50/1
Synthetic rhodomyrtone	0.50/2	2/4	0.125–4	0.25–4	1/4
Erythromycin	0.03/0.03	0.25/0.50	0.03–2	0.03–4	0.125/0.25

**Figure 1:**
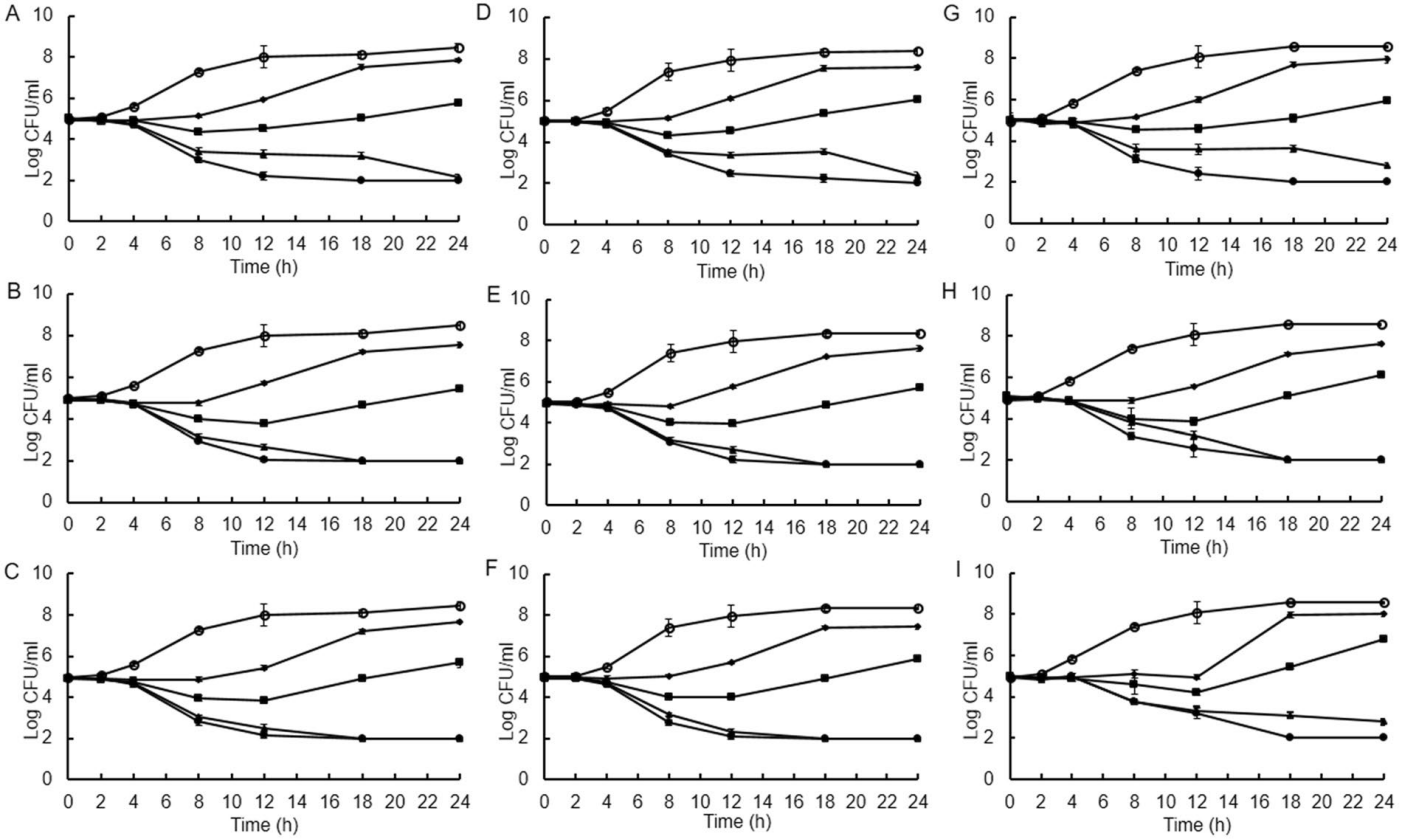
Time-kill curves of *S. pneumoniae* R6 (**A**–**C**), TIGR4 (**D**–**F**) and 5335-5 (**G**–**I**) after treatment with *Rhodomyrtus tomentosa* ethanol extract (**A**,**D** and **G**), purified rhodomyrtone (**B**,**E** and **H**), and synthetic rhodomyrtone (C, F and I) at 4 × MIC (●), 2 × MIC (▲), 1 × MIC (■), and 0.5 × MIC (♦). One percent of DMSO (o) was used as control. The results are shown as mean ± SD of three independent cultures.

To confirm the antimicrobial effectiveness of rhodomyrtone against the pneumococcus, we performed time-kill curves of the three testing preparations at different MICs, using three pneumococcal strains: the reference strains R6 and TIGR4, and the clinical isolate 5335-5, as this presented intermediate MIC/MBC values for rhodomyrtone from the whole collection, but one of the highest MIC/MBC values for erythromycin, a macrolide antibiotic used for treating pneumococcal infections. As shown in Fig. 1, antibacterial activity of the extract and the compounds was concentration dependent, resulting in the reduction of colony forming units. The viability of *S. pneumoniae* after exposure to the extract and the pure compounds at 4 × MIC decreased clearly by 3 logfolds after 18 h for the three tested strains, and even after 12 h for R6 and TIGR4. Furthermore, addition of the extract and the compounds to the culture at 2 × MIC resulted in decreased cell growth. The extract and the compounds at their MIC values exhibited bacteriostatic effects against *S. pneumoniae* whereas 0.5 × MIC had slight effects on the viability of the tested pathogen.

### Effect of purified rhodomyrtone on pneumococcal growth

In order to investigate the effect of rhodomyrtone at proteomic and metabolomic level, we first studied the growth of the reference strains TIGR4 and R6 in the presence of 0.5 × MIC purified rhodomyrtone. As shown in Figure S1, both strains slowly grew in the antimicrobial-treated cultures. Lag phase of the treated TIGR4 and R6 cells was extended to 8 and 14 h after the bacterial cells started to grow, respectively. The treated TIGR4 and R6 cells reached the stationary phase after 12 and 18 h of incubation, respectively. We chose the mid-log phase for sampling proteins and metabolites, in time points such that for each treated and non-treated culture, the cell growth was the same for both. This corresponded to OD_600_ = 0.3 for TIGR4, and OD_600_ = 0.2 for R6.

### Effect of purified rhodomyrtone on the pneumococcal proteome

**Figure 2:**
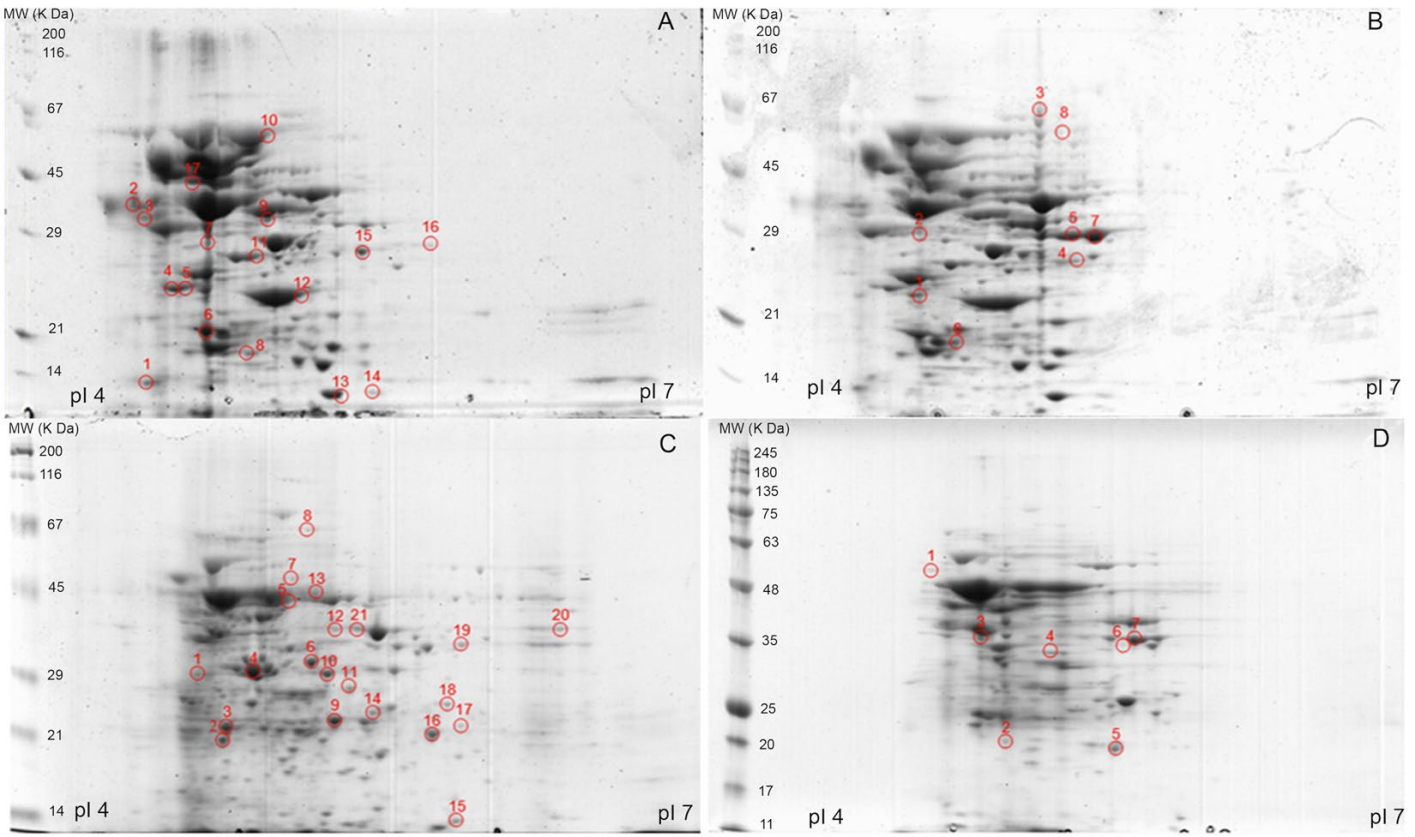
Representative 2-D gels of cellular proteins of *S. pneumoniae* TIGR4 (**A** and **B**) and R6 (**C** and **D**) cultured without (**A** and **C**) and with 0.5 × MIC purified rhodomyrtone (**B** and **D**). The isolated proteins were separated by isoelectric focusing in the pI range of 4 to 7 in the first dimension (11 cm). The proteins were further separated by 10% SDS-PAGE in the second dimension. Spot numbers indicate spots with altered abundances: those marked in (**A** and **C**) diminished after rhodomyrtone treatment, and those marked in (**B** and **D**) augmented after rhodomyrtone exposure.

**Figure 3:**
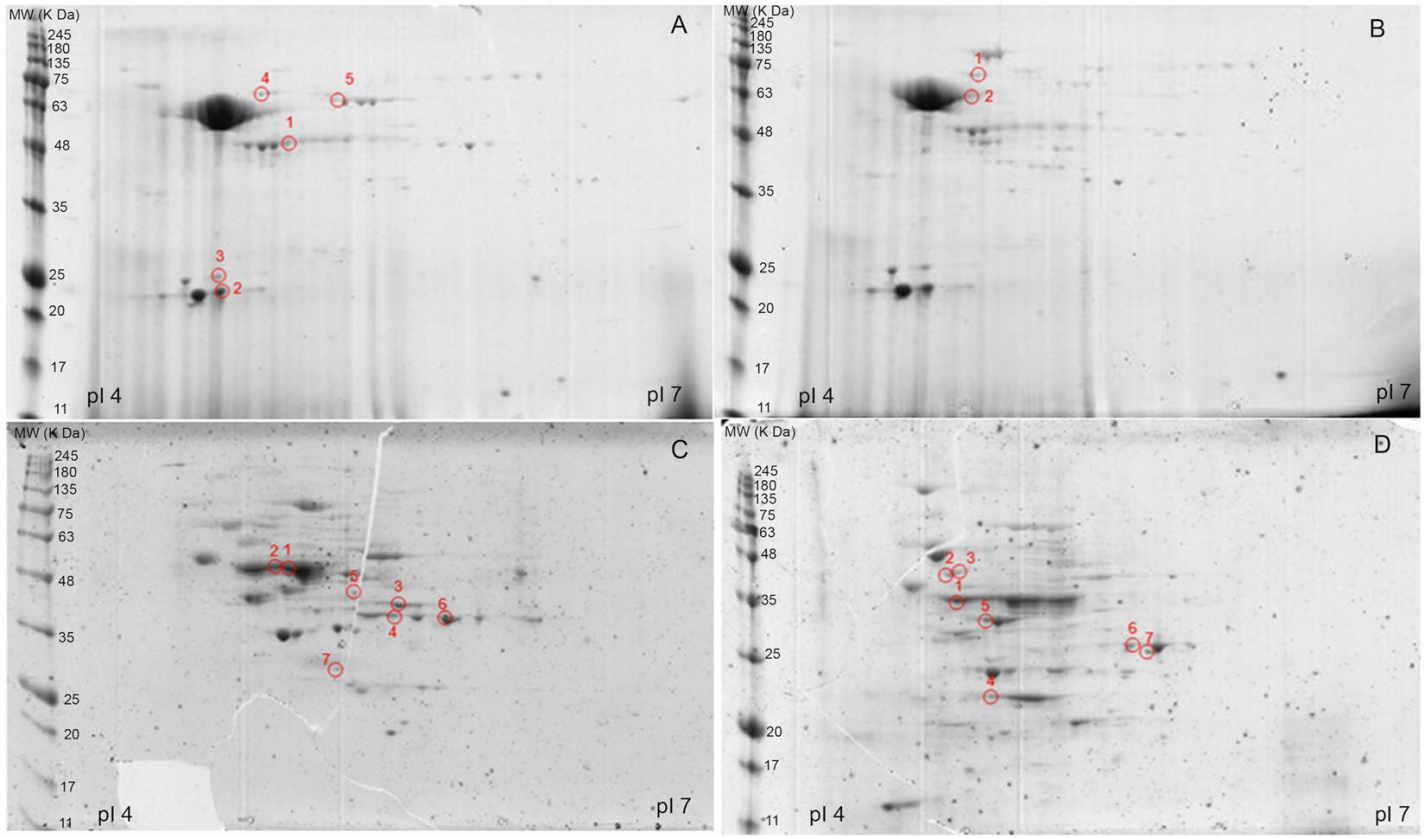
Representative 2-D gels of secreted proteins of *S. pneumoniae* TIGR4 (**A** and **B**) and R6 (**C** and **D**) cultured without (**A** and **C**) and with 0.5 × MIC purified rhodomyrtone (**B** and **D**). The isolated proteins were separated by isoelectric focusing in the pI range of 4 to 7 in the first dimension (11 cm). The proteins were further separated by 10% SDS-PAGE in the second dimension. Spot numbers indicate spots with altered abundances: those marked in (**A** and **C**) diminished after rhodomyrtone treatment, and those marked in (**B** and **D**) augmented after rhodomyrtone exposure.

We analyzed by 2-DE the changes in the pneumococcal proteome of the two reference strains TIGR4 and R6 after rhodomyrtone exposure in two protein fractions, the total cell extract and the secreted proteins. The cellular and secreted protein patterns of both strains are shown in Figs 2 and 3, respectively. Alteration in the abundance of 72 protein spots was observed when *S. pneumoniae* was exposed to 0.5 × MIC purified rhodomyrtone. Sixteen cellular protein spots were decreased, while 8 spots were increased in TIGR4 (Fig. 2A and B). In R6, 20 spots decreased and 7 increased their abundances after rhodomyrtone exposure (Fig. 2C and D). In the secreted fraction, 5 spots of TIGR4 were reduced after rhodomyrtone treatment, while 2 spots increased them (Fig. 3A and B). In the R6 strain, 7 spots increased and other 7 decreased in response to rhodomyrtone (Fig. 3C and D). The selected protein spots were further analyzed by MALDI-TOF/TOF MS. The identified proteins for both cellular and secreted fractions are given in Tables 2 and 3, respectively. The proteins were identified as enzymes involved in important metabolic pathways such as amino acid, carbohydrate, lipid, and nucleic acid metabolism, as well as other factors involved in protein synthesis.

**Table 2:** Cellular proteins altered after purified rhodomyrtone treatment of *Streptococcus pneumoniae*.

Spot #	Accession number^a^	Protein annotation	Mascot score	Coverage (%)	Fold change^b^	*p*-value^c^
4 C	Q8DMY8	Cysteine synthase	180	21	0.36	0.032
10 C	Q8DN31	Arginine deiminase	316	19	0.41	0.018
12 C, 21 C	P65608	Ornithine carbamoyltransferase	158	23	0.31	0.021
4 A	Q97NL1	Glyceraldehyde-3-phosphate dehydrogenase, type I	308	30	0.29	0.019
6 A	P66942	Triosephosphate isomerase	104	24	0.33	0.028
11 A, 6 C, 11 C	P0A3M9, P0A3N0	L-lactate dehydrogenase	101	22	0.44	0.045
14 A	P58313	UTP-glucose-1-phosphate uridylyltransferase	103	31	0.38	0.010
1 C	Q8DRG7	Glycosyltransferase, family 2	98	25	0.30	0.013
16 C	Q8DR15	3-ketoacyl-(acyl-carrier-protein) reductase	95	20	0.37	0.023
7 C	Q8DPM2	DNA gyrase A subunit	96	21	0.40	0.039
5 C	P64031	Elongation factor Tu	517	26	0.29	0.009
6B	Q97SV1	50 S ribosomal protein L5	87	18	1.93	0.034
3D	Q8DN74	Glucose-6-phosphate isomerase	83	19	2.41	0.022
7B	P64022	Elongation factor G	348	24	2.75	0.043

aAccession numbers correspond to UniProt. ^b^The values represent the intensities ratio between rhodomyrtone treatment and control for each spot. Values > 1 indicate an increase in protein abundance. Values < 1 indicate a decrease in protein abundance. ^c^Data were analyzed using the Student’s *t-*test. *P*-values < 0.05 were considered statistically significant.

**Table 3:** Secreted proteins altered after purified rhodomyrtone treatment of *Streptococcus pneumoniae*.

Spot #	Accession number^a^	Protein annotation	Mascot score	Coverage (%)	Fold change^b^	*p*-value^c^
3 C	P65608	Ornithine carbamoyltransferase	355	25	0.38	0.023
5 C	P63414	Acetate kinase	104	23	0.40	0.033
6 C	Q8CWN6	Glyceraldehyde-3-phosphate dehydrogenase	403	32	0.37	0.037
2 C	P64031	Elongation factor Tu	520	29	0.39	0.029
7 C	P0A4S2	Fructose-1,6-bisphosphate aldolase	139	22	0.48	0.047
2D	Q8DPS0	Enolase	396	30	2.54	0.018
5D	Q8DQX8	Phosphoglycerate kinase	848	31	1.97	0.026
7D	Q8DQ85	6-phosphofructokinase	154	26	2.66	0.017
4D	Q8CWV4	50 S ribosomal protein L5	81	20	1.85	0.040
3D	Q6VB96	60 kDa chaperonin	230	29	2.11	0.024

aAccession numbers correspond to UniProt. ^b^The values represent the intensities ratio between rhodomyrtone treatment and control for each spot. Values > 1 indicate an increase in protein abundance. Values < 1 indicate a decrease in protein abundance. ^c^Data were analyzed using the Student’s *t-*test. *P*-values < 0.05 were considered statistically significant.

The combined analysis of both cellular and secreted proteomes revealed that proteins related to protein synthesis, as cysteine synthase and ribosomal proteins, decreased in response to purified rhodomyrtone treatment, as well as the elongation factor Tu, which was strongly reduced. A clear decrease in two out of the three enzymes of the arginine deiminase (ADI) pathway, i.e. arginine deiminase and ornithine carbamoyltransferase was also found. Furthermore, we observed a down-regulation of proteins involved in carbohydrate metabolism. Important enzymes associated with the glycolysis pathway including glyceraldehyde-3-phosphate dehydrogenase, fructose-1,6-diphosphate aldolase, and triosephosphate isomerase were diminished. However, an increase in the levels of glucose-6-phosphate isomerase, enolase, phosphoglycerate kinase, and 6-phosphofructokinase was observed after rhodomyrtone treatment. There was also a decrease in the enzymes L-lactate dehydrogenase and acetate kinase, involved in pyruvate metabolism. A decrease in the levels of 3-oxoacyl-(acyl-carrier-protein) reductase, an enzyme involved in the fatty acid biosynthesis pathway, was also observed in R6 cellular protein fractions, as well as a decrease in the DNA gyrase A subunit, a target for quinolone antibiotics.

A very interesting finding from the proteomic analysis was the decrease in two enzymes taking part in the synthesis of the pneumococcal capsule polysaccharide: the UTP-glucose-1-phosphate uridylyltransferase (GalU), which diminished in rhodomyrtone-treated TIGR4 cellular protein fractions, and the family-2 glycosyltransferase encoded in the locus *spr0136*, which diminished after exposure of R6 to the compound. In both cases, the corresponding protein spots were absent in the cellular-protein 2-D gels.

### Effect of purified rhodomyrtone on the pneumococcal metabolome

The changes of the metabolomic profile of both strains of *S. pneumoniae* treated with the compound were monitored using LC-MS/MS analysis. We carried out a multivariate statistical analysis with the identified compounds to evaluate whether the rhodomyrtone treatment had a significant effect on the metabolite profile. As shown in Figure S2, a principal component analysis (PCA) resulted in a clear distinction between rhodomyrtone-treated and non-treated metabolite fractions for both R6 and TIGR4 strains. The first principal component (PC1, X-axis) was able to completely separate the two samples, i.e. control and rhodomyrtone-exposed cultures for the two strains, grouping the three biological replicates in the same cluster, each analyzed in duplicate. Thus, the PC1 was able to explain most of the variance that was found. This indicates that the rhodomyrtone treatment lead to a clear alteration of the metabolite profile in the two studied pneumococcal strains.

Alteration in the metabolite expression is shown in Table 4. Twenty-six metabolites were detected to change between both control and treatment groups. Eighteen metabolites were found with lower levels after rhodomyrtone treatment, while eight metabolites were more abundant after treatment with the compound. The most highlighting findings of the metabolomic analysis was the alteration of metabolites involved in capsule biosynthesis, in agreement with the results obtained in the proteomic analysis. Three compounds clearly decreased their levels after rhodomyrtone treatment: two in both strains (uridine 5′-diphosphoglucuronic acid, 1.69-fold and 3.0-fold in R6 and TIGR4 respectively; UDP-glucose, 1.31-fold and 1.27-fold in R6 and TIGR4 respectively), and one in TIGR4 only (UDP-N-acetyl-D-galactosamine, 3.12-fold decrease). In addition, other metabolites involved in the synthesis of nucleic acids and amino acids were also altered, in agreement to the fact that some of the pathways were found altered according to the proteomic analysis. Of note, it is worthy to highlight the almost 2-fold decrease in the levels of acetyl-CoA, a key intermediate in different metabolic pathways playing a central role in the primary metabolism.

**Table 4:** Metabolites altered after purified rhodomyrtone treatment of *Streptococcus pneumoniae*.

Metabolite	Strain	MW (g/mol)	Retention time (min)	Precursor ion (m/z)	Fold change^a^	Related pathway or function
Metabolites with decreased abundance						
Thymidine monophosphate	R6, TIGR4	322.057	2.7	321.049	13.49*, 1.71	Nucleic acid biosynthesis
L-Tryptophan	R6, TIGR4	204.090	4.8	205.097	1.34*, 1.70*	Amino acid biosynthesis
Uridine 5′-diphosphoglucuronic acid	R6, TIGR4	580.034	1.1	579.028	1.69*, 3.00*	Capsule synthesis
N-Acetyl-L-glutamic acid	R6, TIGR4	189.064	1.8	188.056	2.11*, 1.09	Urea cycle
UDP-glucose	R6, TIGR4	566.055	1.1	565.047	1.31, 1.27*	Capsule synthesis
Hypoxanthine	R6, TIGR4	136.039	1.6	137.046	1.02, 2.21*	Nucleic acid biosynthesis
Deoxyinosine	R6	252.086	3.7	251.077	INF^b^	Nucleic acid biosynthesis
Guanosine	R6, TIGR4	283.092	2.2	284.099	1.01, INF	Nucleic acid biosynthesis
Inosine	R6	268.081	3.3	267.074	1.07	Nucleic acid biosynthesis
D-(+)-3-Phenyllactic acid	R6, TIGR4	166.063	6.6	165.054	INF, INF	Antimicrobial compound
L-Aspartic Acid	TIGR4	133.038	1.0	132.030	1.35	Amino acid biosynthesis
N-Acetyl-DL-methionine	TIGR4	191.062	5.1	190.054	4.77*	Oxidative stress response
Acetyl-CoA	TIGR4	809.126	5.0	403.556	1.98*	Lipid metabolism, activating intermediate molecule
Raffinose	R6	504.169	1.2	503.162	2.03*	Raffinose/stachyose/melibiose transport system
Deoxyadenosine monophosphate	TIGR4	331.068	1.6	332.075	1.04	Nucleic acid biosynthesis
Palmitic acid	R6, TIGR4	256.240	0.4	257.247	1.09, 1.38	Lipid biosynthesis
UDP-N-acetyl-D-galactosamine	TIGR4	607.082	1.2	606.074	3.12*	Capsule synthesis
Pyroglutamic acid	R6	129.043	1.5	128.035	3.77*	Amino acid metabolism
Metabolites with increased abundance						
Guanosine monophosphate	R6, TIGR4	363.058	1.5	362.052	3.38*, 2.32*	Nucleic acid biosynthesis
L-Tyrosine	R6	181.074	1.8	180.067	1.08	Amino acid biosynthesis
Uridine monophosphate	R6, TIGR4	580.034	1.1	579.028	4.73*, 1.37*	Nucleic acid biosynthesis
D-Ribulose 5-phosphate	R6	230.019	0.7	229.012	1.05	Pentose phosphate pathway
D-Glucose 6-phosphate	R6, TIGR4	260.030	0.7	259.023	1.71, 5.25*	Glycolysis
Cyclic adenosine diphosphate ribose	R6, TIGR4	541.061	1.4	540.055	2.85*, 1.04	Calcium signaling
L-Glutamic acid	R6, TIGR4	147.053	0.7	146.046	1.37, 1.44*	Amino acid biosynthesis
L-Phenylalanine	R6	165.079	3.5	166.086	1.69*	Amino acid biosynthesis

^a^The fold change values represent the ratio between control and rhodomyrtone treatment for metabolites with decreased abundance, or the ratio between the rhodomyrtone treatment and the control for metabolites with increased abundance. Statistical significance of the analysis under the Student’s *t*-test is indicated as *(*p* < 0.05). ^b^INF means that the metabolite was not detected in the rhodomyrtone-treated samples.

### Effect of purified rhodomyrtone on the pneumococcal capsule

The results from both proteomic and metabolomic analyses revealed that purified rhodomyrtone reduced the levels of biomolecules involved in the biosynthesis of the pneumococcal capsule. Hence, we studied the effects of the compound on pneumococcal capsule formation.

**Figure 4:**
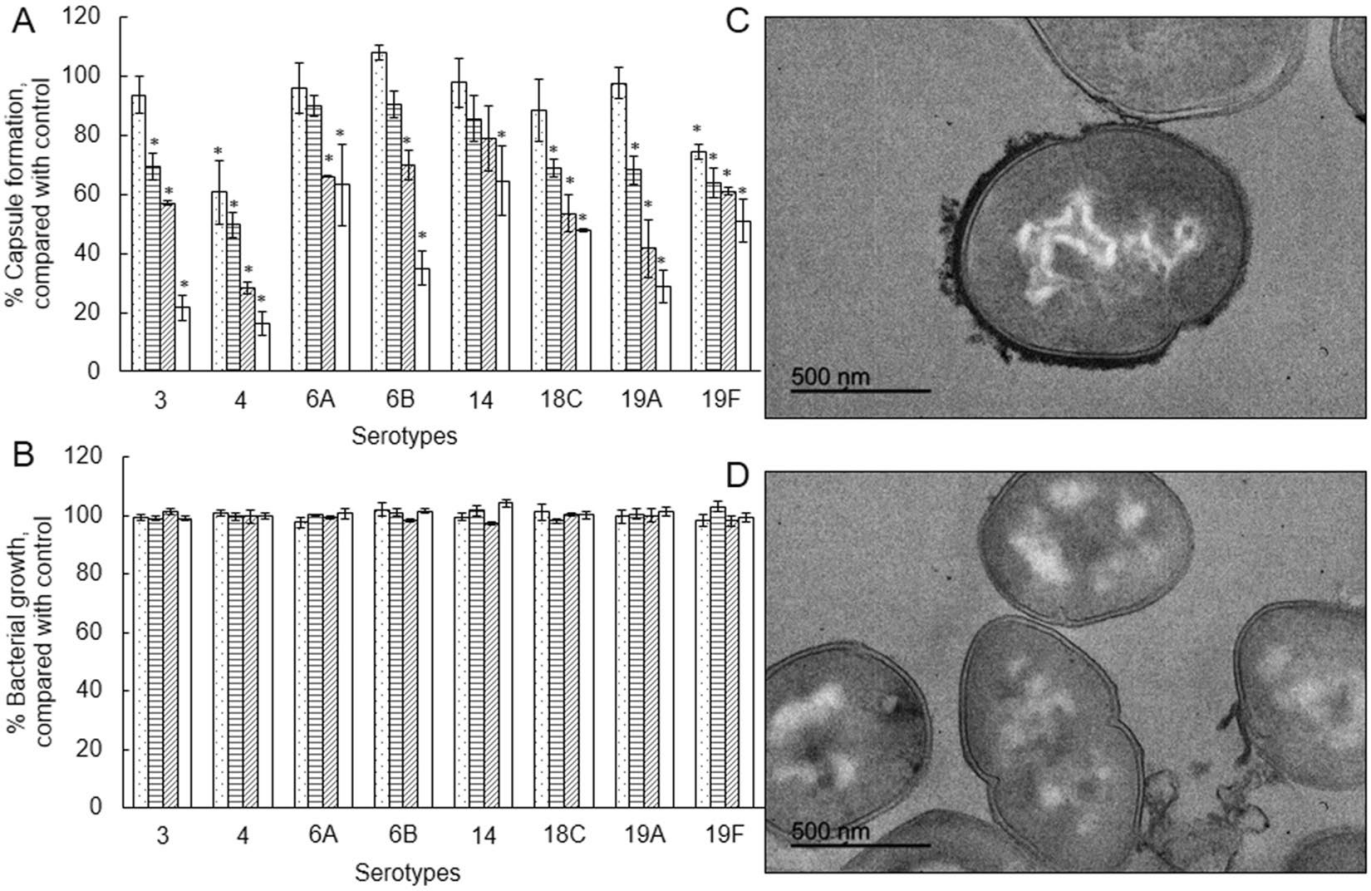
Inhibition of pneumococcal capsule by purified rhodomyrtone. Effects of purified rhodomyrtone at different concentrations including 0.5 × MIC (), 0.25 × MIC (), 0.125 × MIC (), and 0.063 × MIC () on percentage of pneumococcal capsule (**A**) and growth (**B**), compared with control (1% DMSO). The relative percentage of capsule formation was defined as: (mean A640 of treated cells/mean A640 of control) × 100. Capsular illustration by transmission electron microscopy of *S. pneumoniae* TIGR4 cells before (**C**) and after exposure to 0.5 × MIC purified rhodomyrtone (**D**). *p < 0.05.

The inhibitory activity of purified rhodomyrtone on pneumococcal capsule formation was assessed on 8 clinical isolates of *S. pneumoniae* representing 8 serotypes. The amount of pneumococcal capsular polysaccharide produced by the cells treated with sub-MIC rhodomyrtone was quantified by the colorimetric Stains-all assay. The compound caused a reduction of capsule production in a concentration-dependent manner, resulting in a reduction of capsular polysaccharide contents (Fig. 4A). However, the tested sub-MICs rhodomyrtone had no inhibitory effects on the growth of *S. pneumoniae* clinical isolates (Fig. 4B), thus indicating that the lower amount of measured capsule was not due to a lower number of cells grown in the cultures. Rhodomyrtone-treated pneumococcal cells significantly possessed less amount of capsule, when compared with untreated cells. The reduction in capsular polysaccharide contents was greatest when the bacteria were treated with 0.5 × MIC rhodomyrtone. At this concentration, the percent reduction of capsular polysaccharide formation of *S. pneumoniae* serotypes 3, 4, 6 A, 6B, 14, 18 C, 19 A, and 19 F by rhodomyrtone were 75, 80, 33, 62, 43, 53, 67, and 57%, respectively. The highest reduction was observed in serotype 4, with percentages ranging from 29–80%.

We also observed changes, at a qualitative level, in the presence, morphology and/or thickness of pneumococcal capsule in two strains (the reference TIGR4 and the serotype-19A strain 56 H) treated with the compound using transmission electron microscopy. Rhodomyrtone-treated TIGR4 cells clearly possessed less amount of capsule, when compared with untreated cells (Fig. 4C and D, and Figure S3). The rhodomyrtone treatment also seemed to indicate a reduction in the capsule of the strain 56 H when observed by electron microscopy (Figure S4).

## Discussion

Emergence of antimicrobial resistance in pathogenic microorganisms is a global healthcare problem, which has been directly related to the increasing consumption and/or overuse of antibiotics by the population^11, 19^. Particularly, the pneumococcus has become increasingly resistant to the most common antibiotics used to combat this pathogen (i.e. β-lactams, macrolides and quinolones) over the last decades, mainly because of the spread of pneumococcal clones of the so-called “pediatric serotypes”, i.e. 6 A, 6B, 9 V, 14, 15 A, 19 A, 19 F, and 23 F^11^. Actually, it is thought that resistance selection occurs mainly in pneumococci colonizing young children, as they have high carriage rates and exposure to antibiotics, which favors the selection of drug resistance^20^. However, selective pressure by vaccine-induced host immunity may also contribute to the appearance of new resistant strains, by increasing the frequency of serotypes with high non-susceptibility prevalence^21^.

Discovery of new antimicrobials is crucial to fight against possible outbreaks of drug-resistant pathogens. Therefore, several studies have focused on alternative strategies to cure pneumococcal infections^22–24^. It has been known for a long time that plants are a source of antimicrobial compounds^25^. In this research, we have studied the anti-pneumococcal property of rhodomyrtone, an acylphloroglucinol compound obtained from leaves of the Asian South-East plant *Rhodomyrtus tomentosa*. Our results demonstrated that the extract and the compounds (both the purified and the synthetic ones) exhibited strong antibacterial activity against a collection of clinical isolates representative of the most prevalent serotypes circulating in Spain during the last years. We observed that the MIC_90_ of rhodomyrtone for the clinical isolates was 64 times lower than that of the ethanol extract, which confirms it as an effective antimicrobial compound in the ethanol extract of *Rhodomyrtus tomentosa* leaves. It possessed pronounced antibacterial activity against all our pneumococcal isolates, even against those less susceptible to erythromycin, which might indicate differences in the mechanisms of action of both substances. Moreover, our previous studies found that the ethanol extract and rhodomyrtone have a potential as a remarkable antibacterial agent to control a broad range of Gram-positive pathogens^14, 15, 26, 27^. This information indicated that rhodomyrtone presented a narrow-spectrum antibacterial property with low levels of MIC and MBC. In addition, previous studies have shown that the purified compound did not produce toxic effects on human erythrocytes at a concentration of 128 µg/ml^26^.

The Systems Biology era offers new opportunities to study global changes in microbes responding to any stimulus or stress, by means of the overview that the integration of different omic platforms provides^28, 29^, which can be also applied to study the effect of antibiotics^30^. However, there is a general lack of metabolomic studies in bacteria, and particularly in the pneumococcus. In previous works, we have targeted the pneumococcal surface proteome aiming at the discovery of vaccine and/or diagnostic candidates^1, 2, 31, 32^. Very recently, we have studied the effect of iron deprivation in two reference pneumococcal strains, using a combination of transcriptomics, proteomics and metabolomics, and have found correlations when integrating proteome and metabolome information (Jiménez-Munguía *et al*., unpublished results). In the present study, a multi-omics approach was applied to investigate the biological changes in *S. pneumoniae* in the presence of rhodomyrtone.

Proteomic and metabolomic analyses showed the biological changes in protein and metabolite profiles of the two studied pneumococcal reference strains exposed to rhodomyrtone. The altered proteins were enzymes involved in important metabolic pathways. Proteomic analysis of cellular proteins demonstrated that proteins related to protein synthesis including cysteine synthase, ribosomal proteins and elongation factor Tu were reduced in response to the compound, as described for the effects of rhodomyrtone on methicilin-resistant *S. aureus* ^16^. Furthermore, the compound also affected the amino acid synthesis. The levels of aspartic acid and tryptophan were reduced, while glutamic acid, tyrosine, and phenylalanine increased. Very interestingly, two enzymes of the arginine deiminase (ADI) pathway, i.e. arginine deiminase and ornithine carbamoyltransferase clearly decreased. The ADI pathway provides ATP in streptococcal species^33^. Recently, Allan *et al*. have demonstrated the decrease in arginine deiminase after nitric oxid treatment in the pneumococcus, an agent that causes dispersal in bacterial biofilms^34^. Moreover, deletion of the *arcD* gene, located at the ADI operon, impairs *S. pneumoniae* D39 capsule^35^. In addition, enzymes and metabolites involved in carbohydrate metabolism were strongly affected. Some enzymes of the glycolysis pathway were altered by rhodomyrtone, as also observed in *Streptococcus pyogenes* ^18^. In the pneumococcus, the antimicrobial agent linezolid lead to alterations in glycolytic anzymes and lactate dehydrogenase in a similar way to what found in the present work^30^. However, the levels of two glycolytic enzymes (6-PK and Eno) were also altered by a Ru(II) complex X-03^36^, but opposite to what we found, thus suggesting possible differences in the mechanisms of action for the two antimicrobials. Two enzymes involved in the pyruvate metabolism, L-lactate dehydrogenase and acetate kinase, were diminished. It has been reported recently that these two enzymes are altered under oxidative stress^37^, in a condition leading to a decrease in acetyl-CoA as we have found in this work. Also, there was a reduction of DNA gyrase A subunit. This enzyme is the target of quinolone antibiotics^11^. The enzyme 3-oxoacyl-(acyl-carrier-protein) reductase, involved in fatty acid biosynthesis, was also reduced, as described for the antimicrobial agent Ru(II) complex X-03^36^. Nevertheless, it is unclear whether the effect on these described changes is specific or not.

In our view, the integrative proteomic and metabolomic analysis provided the key aspect of this research: rhodomyrtome seems to affect pneumococcal capsule biosynthesis, as revealed by the observation that levels of two enzymes participating in the biosynthetic pathways, i.e. family-2 glycosyltransferase in R6 and UTP-glucose-1-phosphate uridylyltransferase (GalU) in TIGR4, and three metabolites, i.e. UDP-glucose (UDP-Glc), UDP-glucuronic acid (UDP-GlcUA) and UDP-N-acetyl-D-galactosamine (UDP-Gal*p*NAc), clearly diminished. Glycosyltransferases catalyze the assembly of the repeating units of the capsular polysaccharide by transferring of sugar residues from the appropriate sugar donor, i.e. a sugar phosphate, to an activated lipid carrier on the cytoplasmic face of the cell membrane^38, 39^. R6 is a non-encapsulated type-2 strain derived from the encapsulated, virulent D39 strain. R6 lacks a 7,504-bp region of the D39 genome coding for 7 out of the 9 genes (*SPD_0315* through *SPD_0323*) in a cluster responsible for the capsule biosynthesis^40, 41^. However, as in other strains, R6 possesses other loci in the genome annotated as functions participating in exopolysaccharide/capsule synthesis/glycosyltransferase activity which are out of the previously cited cluster region: *spr0091* and *spr0092* code for sugar transferase related protein and capsule polysaccharide biosynthesis protein CapD, respectively; *spr1654* and *spr1655* code for a capsular polysaccharide biosynthesis protein and a glycosyltransferase, respectively; and *spr0135* and *spr0136* code for an exopolysaccharide (EPS) synthesis glycosyltransferase and a glycosyltransferase, family 2, respectively. Whether this last gene product could have a role in the synthesis of type 2 capsule remains unknown, as R6 lacks the genes for capsule formation and, to our knowledge, its function has not been studied so far.

GalU catalyzes the formation of UDP-Glc, which is the substrate for the synthesis of UDP-GlcUA^42^, carried out by the enzyme UDP-glucose dehydrogenase^9^. UDP-GlcUA plays a central role in the formation of many microbial capsules, including those of *S. pyogenes*, *E. coli* K5, and *Cryptococcus neoformans*, as well as of many *S. pneumoniae* serotypes^43^. We tested the effect of the compound at sub-MICs on pneumococcal capsule formation on 8 clinical isolates of *S. pneumoniae* representing 8 serotypes, some of them coinciding with the so-called “pediatric isolates”; i.e. we selected representative isolates of the most prevalent isolates in pediatric patients. Rhodomyrtone-treated pneumococcal cells significantly possessed less amount of capsule, when compared with untreated cells. All the tested isolates were affected, including that of serotype 3. This serotype has a mechanism of capsule biosynthesis different to that of the other serotypes^43^. However, our data revealed that serotype-3 capsule was reduced in the same extension as for the other serotypes. Whether both biosynthesis pathway types are equally affected or not was beyond the objective of this work. Very recently, it has been described that pneumococcal capsule production by strains harboring capsules with acetylated sugars, as for TIGR4, depends on the presence of pyruvate oxidase, and that a type-4 background mutant lacking this enzyme also had much lower levels of acetyl-CoA, suggesting that capsule reduction/loss arises from dysregulation of this crucial metabolite^44^. Pyruvate oxidase converts pyruvate to acetyl-phosphate, which can be converted to acetate via acetate kinase or to acetyl-CoA via phosphate acetyltransferase. Also very recently, it has been reported that a decrease in acetate kinase is correlated to lower acetyl-CoA levels^37^. Therefore, our findings of decreased abundances in both acetate kinase and acetyl-CoA are in agreement with these recently published results.

In addition, other proteins and metabolites may be affected rather than those identified in our analysis, but the fact that using 2-D gels of total cell extract may have masked minor differences that might be present, but undetectable. A question arising from this research is whether the pneumococcal virulence is reduced after rhodomyrtone treatment. We could not see differences in the abundances of virulence factors such as pneumolysin or neuraminidase, but the use of other more sensitive proteomic approaches, rather than 2-D gels, could help to this aim. Further research using mutants in different pneumococcal serotypes will be needed to further resolve the mechanism of action of rhodomyrtone and to study its possible implication in pneumococcal colonization/adherence.

Preliminary data also indicated that rhodomyrtone strongly reduced the amount of extracellular vesicles in >90% (results not shown). We have recently described the production of these surface-derived structures in the pneumococcus^45^, which carry numerous surface proteins and even virulence factors as cargo. Therefore, this work opens a future perspective on the effect of antibiotics on extracellular vesicles.

## Conclusions

The integration of different omics is a powerful tool to shed light into key pathways that can be altered by a given experimental condition. This work provides insight into the effect of rhodomyrtone, a non-conventional antimicrobial compound, on the pneumococcus at the molecular level, by means of integrating proteomics and metabolomics. The data indicated, among other alterations, a reduction of enzymes and metabolites involved in the capsule biosynthesis. These findings indicate that rhodomyrtone has a potential as antibacterial therapy and could be used in the future if resistance to conventional antibiotics used to treat pneumococcal infections emerge significantly. In addition, our study shows the utility of multi-omic approaches to describe the molecular effects of drugs on pathogenic bacteria. Further research is needed to go in depth into the molecular mechanisms of action of rhodomyrtone, which can lead to its use as an alternative antibiotic for the treatment of pneumococcal infections. The study of mutants defective in key enzymes participating in capsule biosynthesis, as revealed in this work, would help to the comprehension of this phenomenon, as well as the study of the surface proteome to understand which surface proteins might be possible targets of this antimicrobial compound.

## Materials and Methods

### Antibacterial agents

Dried leaves of *Rhodomyrtus tomentosa* were ground with a blender, and the powder was extracted with 95% ethanol as described previously, as well as the isolation protocol of rhodomyrtone^14^. The ethanol extract, purified rhodomyrtone, and synthetic rhodomyrtone (Sigma) were dissolved in 100% dimethyl sulfoxide (DMSO).

### Bacterial strains and growth conditions

Twenty-three clinical isolates of *S. pneumoniae* were obtained from Hospital Universitario Infantil Virgen del Rocío (HUIVR), Sevilla, Spain. *Streptococcus pneumoniae* R6, TIGR4, and Hungary 19A-6 were included as reference strains (Table S1). The isolates were maintained on blood agar plates at 37 °C for 24 h with 5% CO_2_, further cultured in Todd-Hewitt broth (THB), and stored in THB containing 30% glycerol at −80 °C until use.

### Determination of minimal inhibitory concentration (MIC) and minimal bactericidal concentration (MBC)

The MIC and MBC values of the ethanol extract, purified rhodomyrtone and synthetic rhodomyrtone against the isolates were determined using a broth microdilution method according to Clinical and Laboratory Standards Institute guidelines^46^. The bacteria were inoculated in Mueller-Hinton broth (MHB) supplemented with 2.5% lysed horse blood, and incubated at 37 °C for 6–8 h with 5% CO_2_. One hundred µl of the bacterial suspension (10^6^ colony forming units/ml, CFU/ml) was added in a 96 well microtiter plate, containing 80 µl of the medium and 20 µl of serially diluted compound, incubated at 37 °C for 18 h with 5% CO_2_. The MIC was expressed as the lowest concentration of the extract/compound that inhibits visible growth after incubation. The MBC was expressed as the lowest concentration of the extract/compound that kills the bacteria. MIC_50_, MIC_90_, MBC_50_, and MBC_90_ were calculated from MIC and MBC values obtained for each isolate.

### Time-kill study

*S. pneumoniae* R6, TIGR4 and 5335-5 were grown in MHB with 2.5% lysed horse blood, supplemented with ethanol extract/compound at concentrations of 0.5×, 1×, 2×, and 4 × MIC, and incubated at 37 °C with 5% CO_2_. The tested medium containing 1% DMSO was included as negative control. Aliquots were removed at different time intervals (0, 2, 4, 8, 10, 12, 18, and 24 h) and serially diluted. Viable bacteria were calculated by plate counts on blood agar, followed by incubation at 37 °C for 24 h with 5% CO_2_.

### Preparation of cellular and secreted proteins

*S. pneumoniae* strains R6 and TIGR4 were cultured in 200 ml of THB/2% choline supplemented with 0.5 × MIC purified rhodomyrtone or 1% DMSO as control, to an OD_600_ = 0.3 for TIGR4 and an OD_600_ = 0.2 for R6, during the time needed to reach the desired OD_600_ value, according to the growth curve (Figure S1). The bacterial cultures were centrifuged at 5,000 × *g* for 10 min to collect culture supernatant (secreted fraction) and cell pellets. Supernatants were filtered using 0.22-µm filters (Millipore), and proteins were precipitated with 10% trichloroacetic acid and placed overnight on ice. The precipitated proteins were further centrifuged at 13,000 × *g* for 30 min. The protein pellets were washed 3 times with ice-cold absolute ethanol. The protein samples were dried and dissolved in rehydration buffer (7 M urea, 2 M thiourea, 4% CHAPS, 0.5% Triton X-100, 0.005% bromophenol blue, 0.5% Bio-lyte 3–10 Ampholytes), and kept at −80 °C until use. To obtain bacterial cellular proteins, the cell pellets were washed twice with PBS/30% sucrose. The bacterial cells were lysed by adding 100 U mutanolysin, incubated at 37 °C overnight, and resuspended in rehydration buffer. The samples were sonicated (6 cycles of 20-s pulses, 90% amplitude), and centrifuged at 5,000 × *g* for 10 min to remove cell debris. Protein-containing supernatants were concentrated using Amicon 10-kDa Ultra-15 Centrifugal Filter Devices (Millipore) according to manufacturer’s instructions, and stored at −80 °C until use. Proteins were cleaned with the 2D- Clean-up kit (GE Healthcare). Protein amounts were quantified by the Bradford assay^47^.

### Two-dimensional gel electrophoresis (2DE)

One hundred μg of protein were subjected to isoelectric focusing (IEF) on 11-cm Immobiline DryStrips immobilized pH gradient (IPG) gel strips (4–7 pH linear gradient (GE Healthcare)). The strips were loaded onto a Bio-Rad Protean IEF Cell system, and IEF was performed at 20 °C using the following conditions: 2 h of passive rehydration, 50 V for 10 h followed by a voltage-ramp (250 V for 15 min; 500 V for 30 min; 2,000 V for 1 h; 6,000 V for 2 h); finally, proteins were focused on 20,000 Vh. For the second dimension, the strips were previously equilibrated in 3 ml equilibration solution (50 mM Tris-HCl pH 8.8; 6 M Urea, 30% glycerol, 2% SDS, 0.002% bromphenol blue) containing 20 mg dithiothreitol and 25 mg iodoacetamide for 15 min at room temperature, respectively. The strips were subsequently placed onto a 1 mm thick 12% polyacrylamide Criterion^TM^ precast gel (Bio-Rad), and covered with warm molten 0.5% agarose. Gels were run at 20 mA/gel until the tracking dye reached the gel bottom. Then, gels were stained with Coomassie brilliant blue G-colloidal solution (Sigma-Aldrich) according to manufacturer’s instructions. Gels were scanned with a GS-800 densitometer (Bio-Rad). Digitized images were analyzed with PD-Quest v8.1.0 (Bio Rad) and the volume spots were used to quantify the differences by calculating the ratio between rhodomyrtone treatment and control. Consistent spots were considered as those whose presence or absence remained constant in overall replicas (3 biological replicates).

### Protein identification by MALDI-TOF/TOF MS

Protein spots of interest were excised from gels and digested automatically employing an Investigator ProPic and ProGest robotic Workstations (Genomic Solutions). Briefly, gel pieces were destained by two washes at 37 °C for 30 min with 200 mM ammonium bicarbonate in 40% (v/v) ACN. Slices were then washed twice, first with 25 mM ammonium bicarbonate for 5 min and later with 25 mM ammonium bicarbonate in 50% (v/v) ACN for 15 min, dehydrated with 100% ACN and finally dried at room temperature for 10 min. Then, 12.5 ng/µl sequence-grade trypsin (Promega) in 25 mM ammonium bicarbonate was added to the gel pieces. Afterwards, the digestion proceeded at 37 °C overnight. Digestion was stopped by adding 10 μl of 0.5% trifluoroacetic acid (TFA); peptides were desalted using µC-18 ZipTip columns (Millipore) and then eluted directly with matrix solution (α-cyanohydroxycinnamic acid at a concentration of 5 mg/ml in 70% ACN/0.1% TFA) onto a MALDI plate using the dry droplet method. The mass spectra were acquired in a 4800 Proteomics Analyzer MALDI-TOF/TOF Mass Spectrometer (Applied Biosystems), in the m/z range from 800 to 4,000, with an accelerating voltage of 20 kV in reflectron mode. Spectra were internally calibrated using peptides from trypsin autolysis ([M + H^+^] = 842.509, [M + H^+^] = 2211.104) with an m/z precision of ± 20 ppm. The most abundant peptide ions were subjected to MS/MS analysis. A combined search (MS plus MS/MS) was performed against UniProtKB/TrEMBL database using MASCOT (Matrix Science Ltd., London) with the following parameters: taxonomy restrictions to *“Streptococcus pneumoniae”*, one missed cleavage, 0.1 Da mass tolerance in MS and 0.2 Da for MS/MS data, Cys carbamidomethylation as a fixed modification and both Met oxidation and Asn/Gln deamidation as variable modifications. Identifications with a Mascot score >70 (*P*-value < 0.05) were considered as significant.

### Preparation of metabolite extracts

*S. pneumoniae* strains R6 and TIGR4 were grown in THB/2% choline with or without 0.5 × MIC of purified rhodomyrtone, incubated at 37 °C with 5% CO_2_ to an OD_600_ = 0.3 for TIGR4 and an OD_600_ = 0.2 for R6, during the time needed to reach the desired OD_600_ value, according to the growth curve (Figure S1). The bacterial cells were harvested by centrifugation at 5,000 × *g* for 7 min at 4 °C followed by two washes with PBS to eliminate residual broth. Subsequently, the pellets were resuspended in PBS/30% sucrose. The bacterial cells were lysed as previously described for preparing cellular proteins. Bacterial metabolites were extracted by adding ice-cold methanol to give a final concentration of 50%. Metabolite-containing supernatants were collected and ultracentrifuged at 105,000 × *g* for 1.5 h, and stored at −80 °C until use.

### Metabolite identification by LC-MS/MS

An Agilent 1200 Series LC system coupled to an Agilent 6540 UHD Accurate-Mass QTOF hybrid mass spectrometer equipped with dual electrospray (ESI) source (Santa Clara, CA, USA) was used. Chromatographic separation was performed using a C18 reverse-phase analytical column (50 mm × 0.46 mm i.d., 3 µm particle size; Teknokroma, Barcelona, Spain), thermostated at 25 °C. The mobile phases were 5% ACN (phase A) and 95% ACN (phase B) both with 0.1% formic acid as ionization agent. The LC pump was programmed with a flow rate of 0.8 ml/min with the following elution gradient: 3% phase B was kept as initial mobile phase constant from min 0 to 1; from 3 to 100% of phase B from min 1 to 13. The injection volume was 3 µl and the injector needle was washed for 10 times between injections with 80% methanol. Furthermore, the needle seat back was flushed for 10 s at a flow rate of 4 ml/min with 80% methanol to avoid cross contamination. The parameters of the electrospray ionization source, operating in negative and positive ionization mode, were as follows: the capillary and fragmentor voltage were set at ± 3.5 kV and 175 V, respectively; N_2_ in the nebulizer was flowed at 40 psi; the flow rate and temperature of the N_2_ as drying gas were 8 l/min and 350 °C, respectively. MS and MS/MS data were collected in both polarities using the centroid mode at a rate of 2.6 spectra per second in the extended dynamic range mode (2 GHz). Accurate mass spectra in auto MS/MS mode were acquired in MS *m/z* range 60–1,100 and MS/MS *m/z* range 60–1,100. The instrument gave typical resolution 15000 FWHM at *m/z* 118.086255 and 30000 FWHM at *m/z* 922.009798. To assure the desired mass accuracy of recorded ions, continuous internal calibration was performed during analyses by using the signals at *m/z* 121.0509 (protonated purine) and *m/z* 922.0098 [protonated hexakis(1 H,1 H,3H-tetrafluoropropoxy)phosphazine or HP-921] in positive ion mode; while in negative ion mode ions with *m/z* 119.0362 (proton abstracted purine) and *m/z* 966.0007 (formate adduct of HP-921) were used. The auto MS/MS mode was configured with 2 maximum precursors per cycle and an exclusion window of 0.25 min after 2 consecutive selections of the same precursor. The collision energy selected was 20 V. MassHunter Workstation software (vesion 5.00 Qualitative Analysis, Agilent Technologies, Santa Clara, CA, USA) was used to process all data obtained by LC-QTOF in auto MS/MS mode. The MSMS METLIN Personal Compound and Database Library (PCDL) was used to identify compounds using both MS and MS/MS information to assure metabolite identification.

### Quantification of capsule

Eight encapsulated *S. pneumoniae* isolates representing 8 serotypes (3, 4, 6 A, 6B, 14, 18 C, 19 A, and 19 F) were used as representative isolates. The amount of capsule was measured using Stains-all assay (Sigma-Aldrich) for detecting acidic polysaccharides as described^48^, with slight modifications. Briefly, the bacteria were inoculated in THB with 5% fetal bovine serum to OD_600_ = 0.5. Then, 5 ml of the bacterial culture was centrifuged at 5,000 × *g* for 10 min, washed twice with PBS and resuspended in 600 µl of normal saline solution (NSS). One hundred µl of the culture was harvested to make dilutions in NSS for plating out to determine the CFU. To quantify acidic polysaccharides, 100 µl of the suspension was added in a tube containing 20 mg 1-ethyl-2(3-(1-ethylnaphthho-(1,2-d) thiazolin-2- ylidene) 2 methylpropenyl) naphthho-(1,2-d)thiazolium bromide (Stains-all) and 60 ml glacial acetic acid in 100 ml 50% formamide. The amount of capsule was determined by OD_640_ measuring. One hundred µl NSS with 2 ml Stains-all solution was used as a blank.

### Transmission electron microscopy

Pneumococcal capsule was observed using transmission electron microscopy (TEM) as described^48^, but without lysin nor acetate in the cacodylate buffer. Briefly, overnight cultures in THB of *S. pneumoniae* TIGR4 were harvested by centrifugation at 5,000 × *g* for 10 min. The bacterial cells were washed twice, resuspended in PBS, and fixed with 2% paraformaldehyde and 2.5% glutaraldehyde in 0.1 M cacodylate buffer (pH 7) containing 0.075% ruthenium red for 20 min on ice. The samples were washed with cacodylate buffer containing 0.075% ruthenium red. The samples were fixed again with the fixing solution for 3 h, washed with cacodylate buffer containing 0.075% ruthenium red, and then post-fixed with 1% osmium tetroxide in cacodylate buffer containing 0.075% ruthenium red for 1 h at room temperature. After dehydration in an ascendant series of ethanol, the pieces were transferred to propylene oxide and sequentially infiltrated in EMbed 812 resin (EMS; USA). We used the sequence propylene oxide-resin 2:1, 1:1, and 1:2 throughout 24 h. Afterwards samples were transferred to pure resin for 24 h. Blocks were formed in fresh resin that was allowed to polymerize for 48 h at 65 °C. After trimming, blocks were sectioned in an Ultracut Reicher ultramicrotome to obtain ultrathin (40–60 nm width) sections using a diamond knife. The sections were observed and photographed in a Jeol Jem 1400 Transmission Electron Microscope at the Servicio Centralizado de Apoyo a la Investigación (SCAI), University of Córdoba.

### Statistical analysis

All the quantitative analyses (proteomics, metabolomics and capsule measurement) were performed from three independent biological replicates, and the results are expressed as the mean ± standard deviation (n = 3). Paired data were analyzed by univariate analysis using the Student’s *t*-test. Principal component analysis (PCA) was done with the web-based software NIA array analysis tool (http://lgsun.grc.nia.nih.gov/anova/index.html)^49^. *p-*Values lower than 0.05 were considered statistically significant.

## Electronic supplementary material

Supplementary Material
