# Supplementary material for: Integrated proteomic and metabolomic analysis reveals that rhodomyrtone reduces the capsule in Streptococcus pneumoniae
Source: Sci Rep. 2017 Jun 2;7:2715. doi: 10.1038/s41598-017-02996-3 (PMC5457420; doi:10.1038/s41598-017-02996-3)
Supplement: Supplementary file 1 — Supplementary Material [file 41598_2017_2996_MOESM1_ESM.pdf]

Integrated proteomic and metabolomic analysis reveals that rhodomyrtone reduces the capsule in *Streptococcus pneumoniae*

Watcharapong Mitsuwan[1], Alfonso Olaya-Abril[2], Mónica Calderón-Santiago[3], Irene Jiménez-Munguía[2], José Antonio González-Reyes[4], Feliciano Priego-Capote[3], Supayang P. Voravuthikunchai[1], Manuel J. Rodríguez-Ortega[2*]

[1]Department of Microbiology and Excellent Research Laboratory on Natural Products, Faculty of Science and Natural Product Research Center of Excellence Prince of Songkla University, Songkhla, Thailand

[2]Departamento de Bioquímica y Biología Molecular, Universidad de Córdoba; Campus de Excelencia Internacional CeiA3, Córdoba, Spain

[3]Departamento de Química Analítica, Universidad de Córdoba; Campus de Excelencia Internacional CeiA3, Córdoba, Spain

[4]Departamento de Biología Celular, Fisiología e Inmunología, Universidad de Córdoba; Campus de Excelencia Internacional CeiA3, Córdoba, Spain

*Corresponding author:

Manuel J. Rodríguez-Ortega

Address: Departamento de Bioquímica y Biología Molecular, Edificio "Severo Ochoa" planta baja, Campus de Rabanales, Universidad de Córdoba. 14071 Córdoba, Spain.

Tel: +34 957 218519

Fax: +34 957 218856

E-mail: mjrodriguez@uco.es

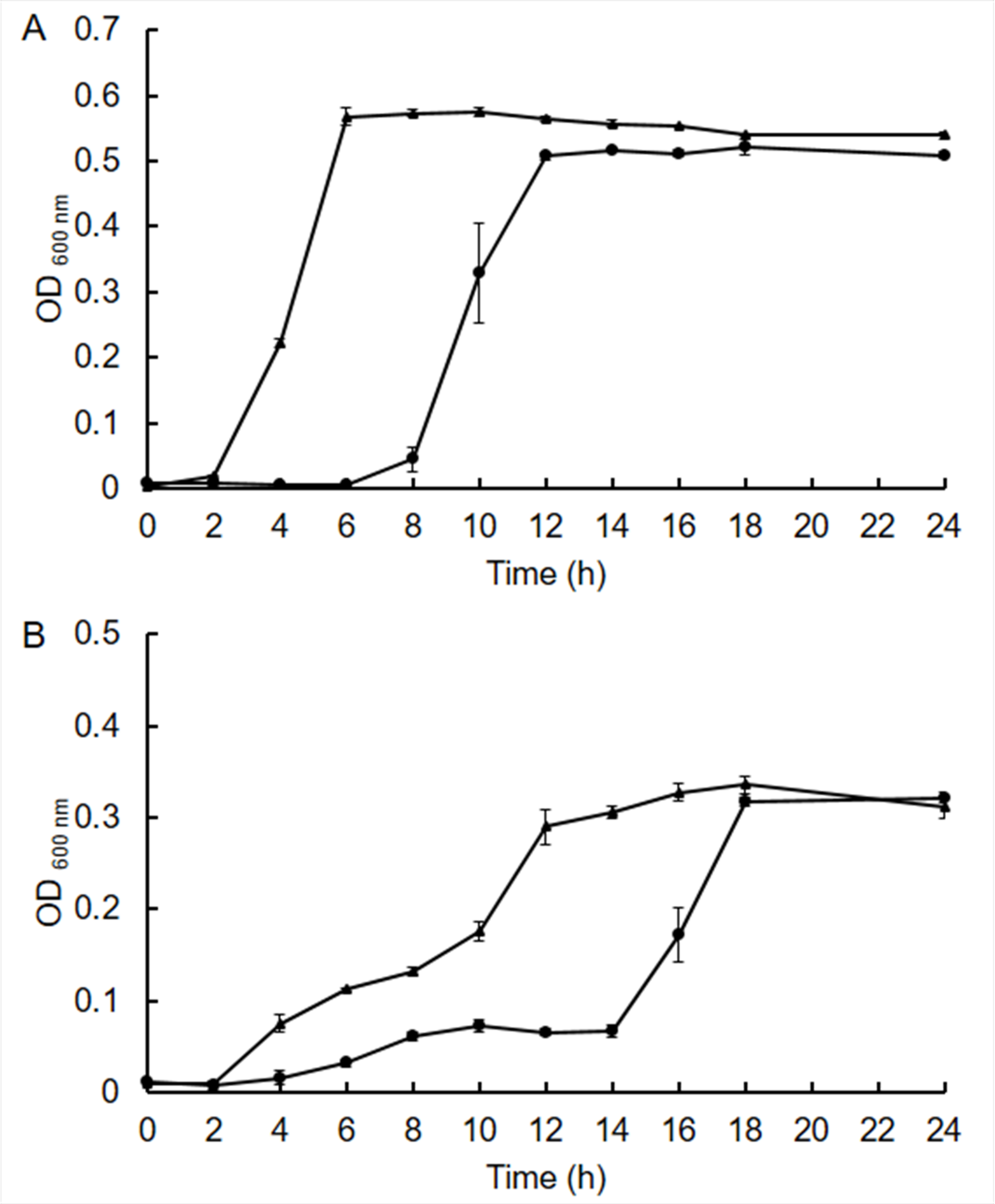

Figure S1. Growth curves of *Streptococcus pneumoniae* strains TIGR4 (A) and R6 (B) in the presence (●) or absence of rhodomyrtone at 0.5 × MIC (▲). 1% DMSO was used as negative control.

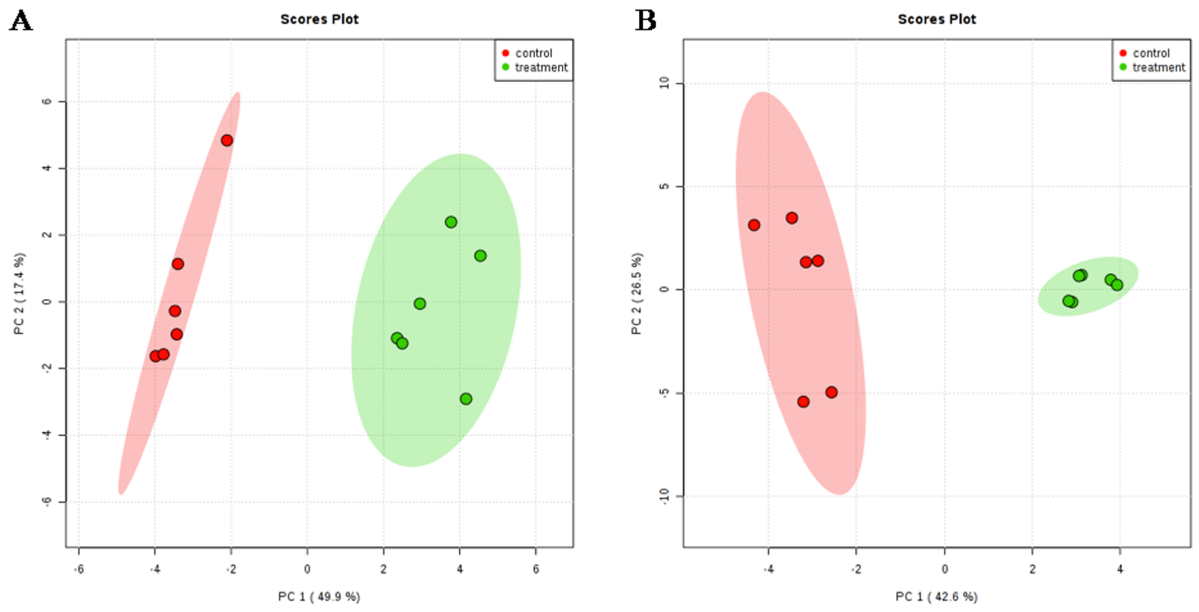

Figure S2. Principal component analysis of the metabolite profile of *Streptococcus pneumoniae* strains TIGR4 (A) and R6 (B) treated with 0.5 × MIC purified rhodomyrtone.

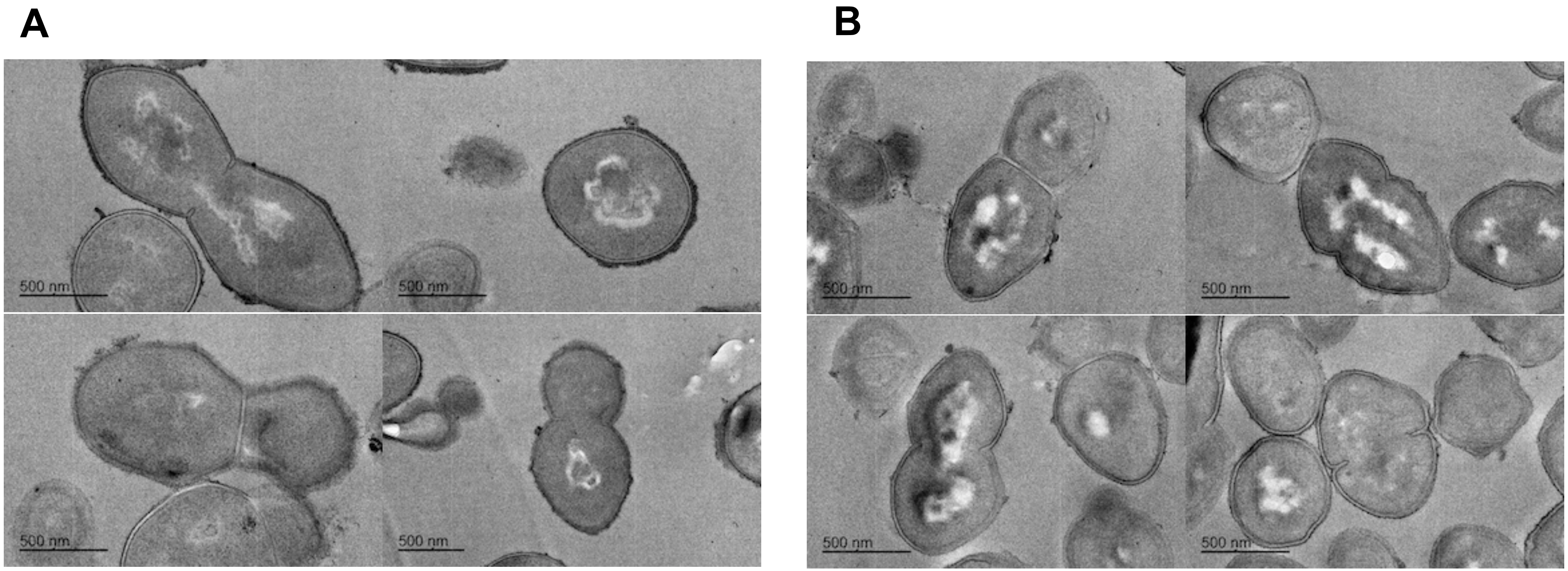

Figure S3. Transmission electron micrographs of *Streptococcus pneumoniae* TIGR4 controls in the absence (A) and in the presence of 0.5 × MIC purified rhodomyrtone (B).

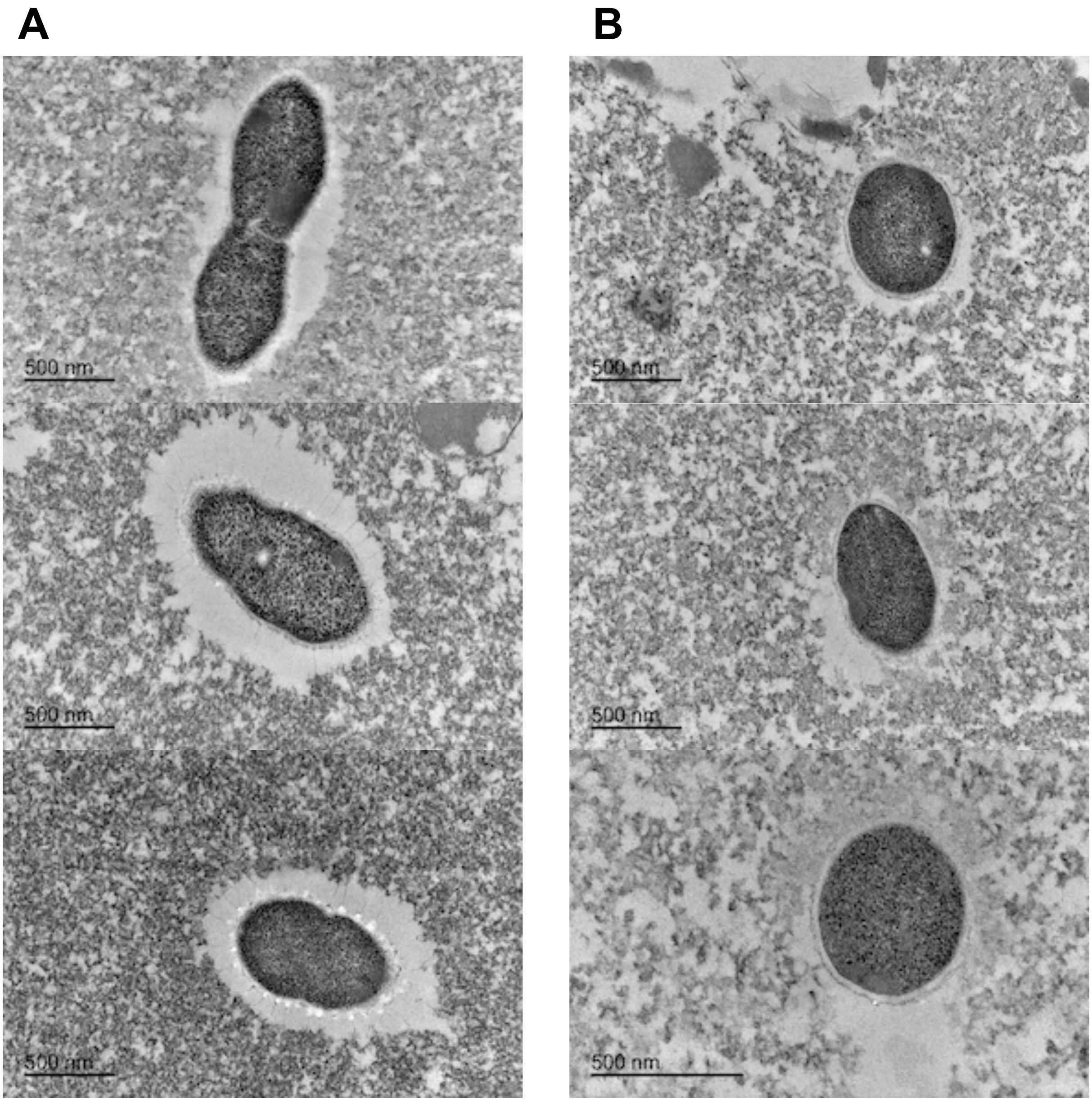

Figure S4. Transmission electron micrographs of *Streptococcus pneumoniae* 56H controls in the absence (A) and in the presence of 0.5 × MIC purified rhodomyrtone (B).

Table S1. Minimal inhibitory concentration (MIC) and minimal bactericidal concentration (MBC) of *Rhodomyrtus tomentosa* ethanol extract, purified rhodomyrtone, and synthetic rhodomyrtone against *Streptococcus pneumoniae* clinical isolates.

Isolates	serotype	origin	MIC/MBC (μg/ml)			
			Ethanol extract	Purified rhodomyrtone	Synthetic rhodomyrtone	Erythromycin
1H	-	-	64/128	2/2	2/2	0.03/0.03
3H	9V	blood	64/128	1/1	2/2	0.03/0.03
6H	23F	blood	64/256	2/4	2/4	2/4
12F	-	-	64/128	1/1	2/4	0.03/0.06
29H	19F	blood	64/256	2/4	2/4	0.25/0.25
49H	19A	blood	256/512	4/4	4/4	0.03/0.125
52H	3	PF	64/512	1/4	2/4	0.25/0.5
56H	19A	blood	32/64	0.5/1	0.5/2	0.06/0.06
56H.2P	-	-	32/128	0.5/2	0.5/2	0.06/0.06
57H	18C	blood	16/16	0.125/0.125	0.125/0.25	0.03/0.03
65H	6B	blood	128/256	2/4	2/4	0.25/1
80H	3	PF	64/256	1/2	2/4	0.06/0.06
83H	1	PF	64/64	2/2	2/4	0.03/0.06

84H	6A	blood	64/128	2/2	2/2	0.03/0.06
87H	18C	CSF	32/64	0.5/1	0.5/1	0.03/0.03
89H	-	-	128/512	4/4	4/4	0.03/0.06
99H	7	blood	64/128	2/4	2/4	0.125/0.125
5104-8	8	-	64/128	1/2	1/2	0.03/0.06
5278-14	14	-	64/256	0.5/1	0.5/1	0.125/0.125
5334-8	8	-	128/512	2/4	2/4	0.03/0.06
5335-5	5	-	64/64	2/2	2/2	0.5/0.5
5335-19A	19A	-	64/128	0.5/0.5	0.5/0.5	0.03/0/03
5533-19A	19A	-	64/256	0.5/0.5	0.5/0.5	0.03/0.03
TIGR4	4	-	256/512	2/8	4/4	0.25/0.25
R6	2	-	64/256	1/2	2/4	0.25/1
ATCC700763	19A-6		32/128	0.5/1	1/4	0.125/0.25
